# Effects of Temperature on the Compressive Strength Parallel to the Grain of Bamboo Scrimbe

**DOI:** 10.3390/ma9060436

**Published:** 2016-06-02

**Authors:** Yong Zhong, Hai-Qing Ren, Ze-Hui Jiang

**Affiliations:** 1 Research Institute of Wood Industry, Chinese Academy of Forestry, Beijing 100091, China; renhq@caf.ac.cn; 2 International Center for Bamboo and Rattan, Beijing 100102, China; Jiangzehui@icbr.ac.cn

**Keywords:** compressive strength, bamboo scrimber, temperature

## Abstract

The objective of this study was to investigate the compressive strength parallel to the grain of bamboo scrimber during and after exposure to various temperatures, in a range from 20 to 225 °C. These data were used to provide a basis for the evaluation of the fire performance of bamboo structures. A total of 152 specimens, assembled as group “during-fire” and “post-fire”, were tested during and after exposure to high temperatures. The experimental results indicated that there were significant differences in compressive properties between the “during-fire” and “post-fire” groups. At one temperature level, the compressive strength and modulus of elasticity of the “post-fire” group were significantly higher than those properties of the “during fire” group, but the ductility coefficient was reversed. FTIR analysis results showed that 175 °C was a key turning point, at which thermal decomposition occurred in the cellulose of the bamboo and phenolic resin.

## 1. Introduction

Employed as a popular green building material, bamboo offers a high strength-to-weight ratio, but is also a rapidly growing plant, which can be harvested as a mature commercial material in three to five years [1,2]. Consequently, bamboo has become increasingly popular throughout the world for use in construction, particularly in China. This is more than a coincidence; rich bamboo resources in China amount to more than 500 species, which represents about one-third of the world’s bamboo.

To fully utilize these abundant resources, many bamboo-based composites such as bamboo scrimber, laminated bamboo lumber and glued-laminated bamboo have been developed in China. These products offer the advantages of high manufacturing efficiency and utilization as well as good physical and mechanical properties [3]. With an increased use of bamboo-based composites, it is important to investigate their combustion behavior during and after exposure to high temperatures, due their relative flammability.

The effect of temperature on wood or wood-based composites has been extensively studied. At high temperature, the physical and mechanical properties of wood are strongly related to the thermal degradation of the polymers in the material and the variation of water content in the product during a fire. There are two traditional methods used to assess the thermo-mechanical properties of wood. One method concerns the immediate effect of temperature where the sample is initially heated to a prescribed temperature and then maintained at this temperature during a mechanical test. Gerhards [4] has summarized the relevant studies on the immediate effect of moisture content and temperature on the mechanical properties of clear wood. For instance, paricá wood loses about 65% of its compressive strength parallel to the grain when exposed to 230 °C heat for 3 h [5]. At 220 °C over a period of 2 h, the embedding strength of *pinus sylvestris* decreased to nearly half its original strength at room temperature [6].

The second method used to ascertain the permanent effect of temperature on wood entails first heating the sample to the prescribed temperature and then cooling it to room temperature, where the mechanical test is performed. Many research studies have employed this method to assess the post-fire residual strength of structural wooden members [7,8,9].

Other than wood or wood-based composites, few studies have been conducted to determine the changes in the mechanical performance of bamboo scrimber at or after exposure to high temperatures. Because of the increased use of bamboo scrimber as a building material, more research on this material is needed. Determination of the performance and mechanical properties of full-sized bamboo structural members, when subjected to extreme heat, is a difficult task to accomplish due to the high cost and complexity of the material and testing. As a result, thermo-mechanical testing of smaller sample materials, to determine its mechanical characteristics at high temperature, is used to assess the safety of structural members exposed to fire [4].

Therefore, the objective of this study was to investigate the compressive strength of bamboo scrimber during and after exposure to various temperatures, to provide a basis for the evaluation of the behavior of bamboo structures in a building fire.

## 2. Materials and Methods

### 2.1. Materials

For this study, 10 pieces of commercial bamboo scrimber plates, fabricated using hot-pressing technology, were supplied by Sichuan Hongya bamboo Co., Ltd. with the dimensions of 20 × 1250 × 2500 mm^3^ (Hongya, Sichuan, China). The raw material of the bamboo scrimber was Ci bamboo (*Neosino calamus affinis*) from Hongya county, Sichuan Province, in the southwestern part of China; harvested at the age of four to five years. The adhesive used to prepare the scrimber plates was a commercially available low molecular weight phenol formaldehyde resin (PF16L510, Beijing Dynea Chemical Industry Co., Ltd., Beijing, China). The resin had a 49% solids content, a viscosity of 20–40 centi Poise (CPS), a pH of 10–11 and was dissolved in water. All the test specimens were randomly cut from these plates and then were used for the determination of the influence of temperature on compressive strength parallel to grain.

The effects of nine temperature levels (Table 1), in the range from 20 to 225 °C, on the compressive strength parallel to grain, were investigated. Eighteen specimens were tested at each temperature level: eight specimens were tested for compressive strength in group A, eight specimens for compressive test in group B, and the other two specimens were subjected to Fourier transform infrared spectroscopy (FTIR) testing.

The dimensions of each specimen were 20 × 20 × 30 mm^3^. Before the compression test, all the specimens were conditioned at 20 °C and 65% relative humidity in a standard room, to arrive at equilibrium moisture content (EMC). The average moisture of all the specimens was 8.2%. The average density and standard deviation were 1.15 and 0.047 g/cm^3^.

### 2.2. Specimen Heat Processing

For each temperature level, 18 specimens were preheated in a drying oven for 120 min, to arrive at the prescribed temperature. After this preheating process, eight specimens were transferred to a temperature-controlled universal testing machine and were maintained at temperature for 5 min, before the compressive strength was determined. These specimens were defined as group A. The other 10 specimens were cooled to room temperature for 24 h directly after the preheating period. The cooled specimens did not require a temperature re-equilibration. After the cooling period, eight specimens of this group were tested to determine the permanent compressive strength and were defined as group B. The other two specimens were used for Fourier transform infrared spectroscopy (FTIR) test.

### 2.3. Mass Loss Test

According to Chinese national standard [10], the mass loss after high temperature calibration can be calculated using Equation (1).

(1) $$m=100(w_{1}-w_{2})/w_{1}$$

where *m* is the mass loss (%), *w*_1_ is the initial mass of the specimens before heating (g), *w*_2_ is the mass of the specimens before the compressive test (g).

### 2.4. Mechanical Test

Static compression tests for the specimens were conducted using an INSTRON 5582 universal testing machine with a temperature-controlled chamber according to Chinese national standard [11]. The load bases were placed inside the chamber. Specimens were loaded at a rate of 1 mm/min, which were continued until failure. The compressive strength parallel to grain (*f*), modulus of elasticity (*E*), and ductility coefficient (μ) were determined using Equations (2)–(4).

(2) $$f=F_{\max}/A$$

**Figure 1:**
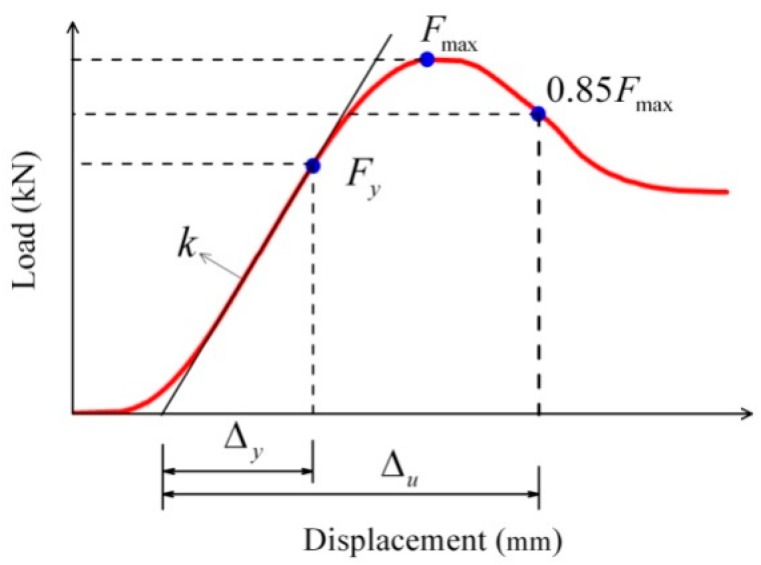
Statistical parameters of compression test.

(3) E=kl/A

(4) $$\mu =\Delta_{u}/\Delta_{y}$$

where *F*_max_ is the maximum load (N), *A* is the cross-section area of the test specimen (mm^2^), *k* is the linear stiffness (N/mm), *l* is the length of specimen (mm), Δ*_u_* is the ultimate displacement (mm), and Δ*_y_* is the yield displacement (mm). These statistical parameters are defined in Figure 1.

### 2.5. FTIR Test

The bamboo scrimber specimen was ground into a powder which was then mixed with the KBr to form a pellet. Fourier transform infrared (TENSOR27, Bruker Corporation, Saarbrucken, Germany) spectra were obtained on the KBr pellet, to determine the relationship between chemical constituents and the mechanical properties of bamboo scrimber.

### 2.6. Statistical Analysis

The graphical analysis was conducted using the Origin 9 software (OriginLab Corporation, Northampton, MA, USA). The analysis of variance (ANOVA) method using SPSS 19.0 (IBM SPSS Corporation, Chicago, IL, USA) was used to analyze the difference of compressive properties between various temperatures, and between the immediate and permanent compressive tests. Multiple comparisons for various temperatures were calculated using the Duncan method. The significance level was set to 0.05.

## 3. Results and Discussion

### 3.1. Mass Loss

**Figure 2:**
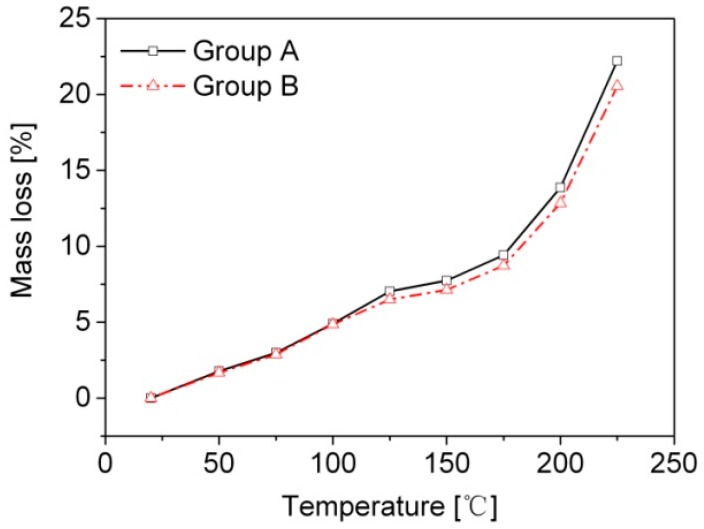
Mass loss of specimens.

The mass losses of the bamboo scrimber samples during and after high temperature testing were measured (Figure 2). The difference in mass loss between group A and B was small, which could be neglected. The rate of mass loss was still low over the temperature range of 125–175 °C. This indicated that the small loss can be mainly attributed to the loss of water; the mass of the bamboo scrimber remained constant when the temperature was below 175 °C. However, the mass loss began to increase rapidly when the temperature grew above 175 °C. At 225° C, the mass loss reached 21%. Zhang *et al.* [12] and Hakkou *et al.* [13] reported on the influence of high temperature on bamboo and hard wood species and found similar results.

### 3.2. Color Changes

**Figure 3:**
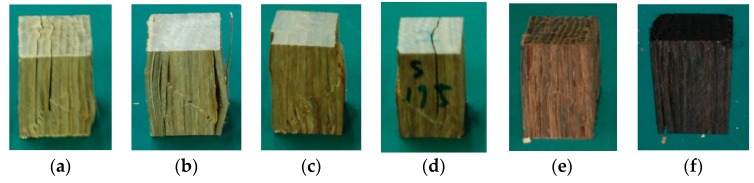
Color changes: (**a**) 20 °C; (**b**) 125 °C; (**c**) 150 °C; (**d**) 175 °C; (**e**) 200 °C; (**f**) 225 °C.

As an indicator of degradation, color change was measured. For both group A and B, during the heating period, a change in the color of the bamboo scrimber occurred in the inner and external areas of the specimens, as determined by visual observation (Figure 3a–f). When the temperature was greater than 150 °C, the changes in color were quite apparent. At 225 °C, the color of the specimens changed to black from light yellow (Figure 3f).

The extent of the color change of the bamboo depended on the temperature to which the sample was exposed [5]. At low temperatures ranging from 50 to 125 °C, the color changes were slight. This is because the major alteration in the product was caused by a loss of water and volatile organic compounds, which lead to physical changes [14]. The color changes are significant at high temperatures, due mainly to the transfer of carbohydrates, phenols and other extracts from the interior of the sample to the exterior during the evaporation of moisture [15,16].

### 3.3. Compressive Analysis

The mechanical properties of bamboo at high temperature change with exposure to heat. Due to this, the compressive strength was measured. The mean values and standard deviations for *f*, *E*, and μ of the test samples are given in Table 2, together with the multivariance analysis. At a set temperature, both the compressive *f* and *E* of group B were significantly higher than those of group A, but the μ of group B was significant lower than that of group A.

**Figure 4:**
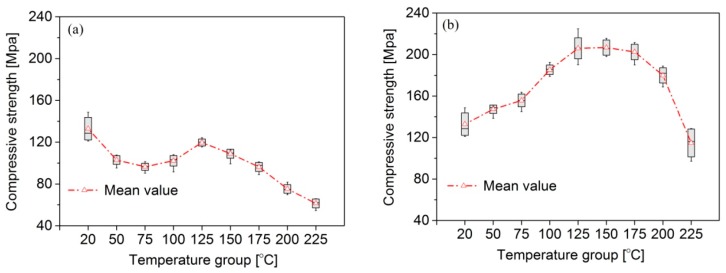
Compressive strength (*f*) of bamboo scrimber exposure to heat: (**a**) group A; (**b**) group B. The box plot shows the mean value, ±1.0 SD (box), Min-Max (whisker).

The compressive strength *f* is influenced by the temperature increase (Figure 4). It contains many complexly influencing factors, such as the moisture content, temperature, and adhesive and raw material itself. For group A (Figure 4a), the compressive strength initially decreased and then increased in the temperature range of 20 to 125 °C. These changes could be the result of two factors. First, some moisture was removed, which has an inverse relation to the strength and can lead to the increase in strength. Second, the softening effects of the high temperature caused *f* to decrease. The findings of Schaffer [17] showed that hemicelluloses soften at 55 °C and lignin begins to soften near 100 °C. Above 125 °C, the compressive *f* decreased linearly with temperature, due primarily to the decomposition of polymers in the wood or to changes in the glass transition temperature of the polymers. As reported by some works [18,19], the hemicelluloses degrade at temperatures between 150 and 200 °C, while lignin decomposes at temperatures between 220 and 250 °C. The hemicelluloses have a glass transition temperature (*T*_g_) in the range of 190–220 °C and lignin has a *T*_g_ in the range of 124–193 °C [20]. In addition, the degradation of the phenol formaldehyde resin occurred at a temperature above 175 °C. According to the variance analysis, there were significant differences in the compressive strength at 20 °C and at other temperatures. After 2 h at 225 °C, the maximum decrease in *f* was 54%, which was consistent with the results reported in the literature [1,5].

For group B, the compressive *f* first increased and then decreased (Figure 4b). There were significant differences in the *f* behavior between groups A and B as a function of temperature. This is because the softening effect of high temperature on the specimens in group B was eliminated when the specimens had been cooled to room temperature, before the compression test. The mean values of *f* for group B were 0.61, 0.72, 1.10 and 0.87 times higher than those for group A at 75, 125, 175 and 225 °C, respectively (Table 2). After 2 h at 225 °C, the bamboo scrimber of group B exhibited a drop of 14% from its initial strength at 20 °C.

**Figure 5:**
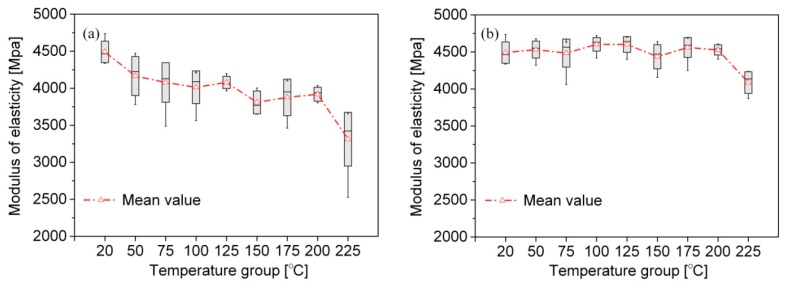
Compressive modulus of elasticity (*E*) of bamboo scrimber exposure to heat: (**a**) group A; (**b**) group B.

The compressive *E* of the tested bamboo scrimber samples is shown in Figure 5. For group A (Figure 5a), the *E* decreased with the increase of temperature mainly because of the softening effects of high temperature. Above 200 °C there was a significant decrease in the *E* due to the degradation of lignin and the glass transition of hemicelluloses [20]. Based on the variance analysis, there were significant differences between the *E* at 20 °C and at other temperatures. The mean value at 225 °C corresponded to 74% of the *E* at 20 °C.

However, for group B (Figure 5b), the bamboo scrimber showed no significant change in the *E* in the range from 20 to 200 °C. There was a significant decrease in the *E* at 225 °C, which was similar to that of group A. The maximum reduction in the *E* was 9%. Zhang *et al.* [12] and Qin [21] reported that the *phyllostachys pubescen* bamboo had a significant decrease in its *E* of up to 200 °C, and attained a reduction of 20% above this temperature. In addition, it is very apparent from these results that the compressive *E* of group B was higher than that of group A (Table 2).

**Figure 6:**
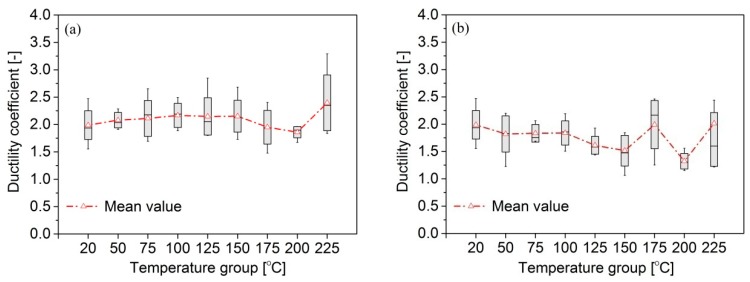
Compressive ductility coefficient (μ) of bamboo scrimber exposure to heat: (**a**) group A; (**b**) group B.

The ductility coefficient (μ) of the treated bamboo scrimber is shown in Figure 6, which represents its plastic deformation capacity. A slight change was observed in μ when the temperature was increased from 20 to 200 °C. The minimum and maximum μ values were 1.86 for 200 °C and 2.40 at 225 °C. According to the variance analysis, there was no difference between the μ at various temperatures except at 225 °C.

Figure 6b shows that the μ of group B decreased slightly at temperatures ranging from 20 to 150 °C. The mean value was 1.52 at 150 °C, and 0.76 at 20 °C. In addition, the μ of group B was significantly lower than that of group A (Table 2), except at 175 °C. At 75, 150 and 225 °C, the mean values of the μ of group B were 0.87, 0.71 and 0.72 times, respectively (Table 2), compared with group A.

### 3.4. Changes of Chemical Components

**Figure 7:**
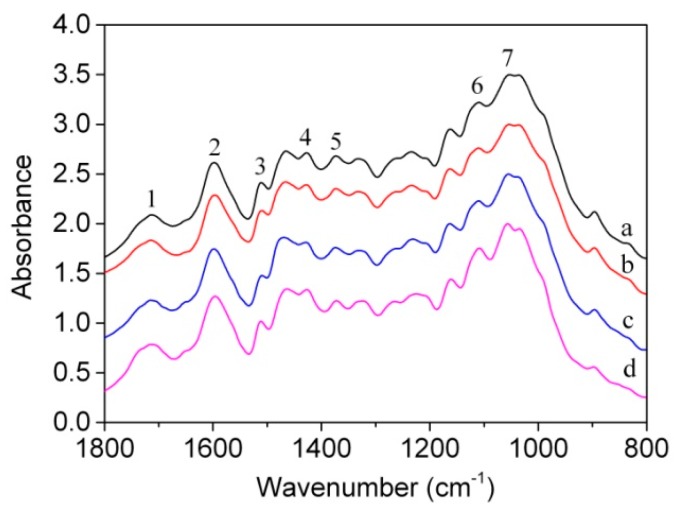
FTIR spectra of bamboo scrimber after explored different temperatures: (**a**) 20 °C; (**b**) 125 °C; (**c**) 175 °C; (**d**) 225 °C.

Based on the previously detailed analysis, obvious changes occurred in the mechanical properties of bamboo scrimber when exposed to temperatures of 125 and 175 °C. Therefore, specimens treated at 20, 125, 175 and 225 °C were selected for FTIR spectrum analysis. The bamboo scrimber was composed of bamboo and phenol formaldehyde resin, which mainly include cellulose, hemicelluloses, lignin and phenolic resin. Figure 7 shows the FTIR spectra of the bamboo scrimber treated at various temperatures. The characteristic bands of the infrared spectra of bamboo scrimber are shown in Table 3.

To determine the changes of chemical components at various temperatures, the absorption peak of bamboo scrimber at 897 cm^−1^ was considered as being characteristic of bamboo. The absorption peak at 1596 cm^−1^ was considered typical of phenolic resin. The relative intensities of C-O and C1 stretching against typical bands are shown in Table 4. These results indicate that both the I_1596_/I_1108_ and I_897_/I_1108_ decreased as the temperature increased. There was a significant difference between the materials at 125 and 175 °C. These results indicated that the temperature of 175 °C is a key turning point for the material at which of the cellulose in the bamboo and the phenolic resin thermally decomposed.

## 4. Conclusions

This investigation focused on determining the mechanical properties of bamboo scrimber subjected to elevated temperatures simulating a building fire. The results of this study led to the following conclusions:

- The average compressive strength parallel to the grain of bamboo scrimber was found to be 133 MPa at 20 °C, 61.4 MPa in a designated “during fire” group and 115 MPa in a “post-fire” group at 225 °C. The strength loss of these two groups was 54% and 14%.
- There were significant differences in the compressive properties between the two groups. At one set temperature level, the compressive strength and modulus of elasticity of the “post-fire” group were significantly higher than the ‘during fire’ group, but the ductility coefficient of these two groups was reversed.
- The compressive strength was affected by temperature, but there were other complicating factors at play including moisture content, the adhesive used and raw material.
- Changes in the color of bamboo scrimber occurred in the inner and external areas of the samples after being subjected to high temperature, which became more apparent at temperatures above 150 °C.
- FTIR test results showed that 175 °C was a key turning point, at which the cellulose in the bamboo and the phenolic compounds in the resin thermally decomposed. These results may help to estimate the behavior of bamboo structures during a fire using numerical analysis, to predict the residual load capacity of the material.

## Figures and Tables

**Table 1:** Average density of heated bamboo scrimber and standard deviation (SD) of compressive tests.

Temperature (°C)	Number of Specimens	Group A		Group B	
		Avg (g/cm^3^)	SD (g/cm^3^)	Avg (g/cm^3^)	SD (g/cm^3^)
20	10	1.117	0.071	-	-
50	18	1.142	0.033	1.173	0.022
75	18	1.167	0.041	1.151	0.052
100	18	1.150	0.025	1.148	0.024
125	18	1.134	0.032	1.141	0.042
150	18	1.143	0.036	1.169	0.042
175	18	1.142	0.034	1.116	0.044
200	18	1.165	0.041	1.153	0.043
225	18	1.144	0.037	1.119	0.061
Total	152				

**Table 2:** Average compressive properties and Duncan test results of bamboo scrimber exposed to heat.

Heated Temperature (°C)	Group A			Group B		
	*f* (MPa)	*E* (MPa)	μ	*f* (MPa)	*E* (MPa)	μ
20	133.0 ± 10.8a	4492 ± 145a	1.99 ± 0.26b	133.0 ± 10.8d	4492 ± 145ab	1.99 ± 0.26b
50	102.9 ± 4.1d	4165 ± 264b	2.08 ± 0.14ab	147.3 ± 4.1c	4532 ± 114ab	1.82 ± 0.33ab
75	96.5 ± 3.4e	4078 ± 268bc	2.11 ± 0.33ab	155.8 ± 6.1c	4484 ± 190ab	1.83 ± 0.16ab
100	102.2 ± 5.0d	4014 ± 221bcd	2.17 ± 0.22ab	185.3 ± 4.6b	4603 ± 91a	1.84 ± 0.22ab
125	119.7 ± 3.3b	4078 ± 83bc	2.15 ± 0.34ab	206.0 ± 10.1a	4602 ± 107a	1.61 ± 0.16bc
150	109.0 ± 4.3c	3810 ± 155d	2.15 ± 0.29ab	206.7 ± 7.4a	4436 ± 163b	1.52 ± 0.28bc
175	96.3 ± 4.3e	3878 ± 247cd	1.95 ± 0.31b	202.5 ± 7.5a	4561 ± 135ab	1.99 ± 0.44a
200	75.3 ± 4.1f	3920 ± 94bcd	1.86 ± 0.10b	179.9 ± 7.3b	4528 ± 69ab	1.32 ± 0.14c
225	61.4 ± 4.3g	3313 ± 363e	2.40 ± 0.51a	114. 8 ± 13.4e	4087 ± 147c	1.72 ± 0.49ab

The letters in the same column represent statistical differences at a 95% confidence level (*p* ≤ 0.05); *f*: compressive strength parallel to grain; *E*: modulus of elasticity; μ: ductility coefficient.

**Table 3:** Characteristic bands of the infrared spectra of bamboo scrimber.

Number	Wavenumber (cm^−1^)	Absorption Peak Location and Assignment
1	1712	C=O stretching of xylan (hemicelluloses)
2	1596	C=C stretching of benzene ring (phenolic resin)
3	1510	Carbon skeleton stretching of benzene ring (lignin of bamboo)
4	1427	C-H stretching of methlene
5	1373	C-H deforming and stretching (cellulose and hemicelluloses of bamboo)
6	1108	C-H stretching of aromatic ring (phenolic resin)
7	1053	C-O stretching
8	897	Anomeric carbon (C1) stretching (cellulose of bamboo)

**Table 4:** Relative intensities of C-O and C1 stretching against typical bands and Duncan test results.

Temperature (°C)	I_1596_/I_1108_	I_897_/I_1108_
20	4.98a	0.93a
125	4.95a	0.84a
175	4.41b	0.61b
225	2.92c	0.25c
